# Precursor B-Cell Acute Lymphoblastic Leukemia/Lymphoma with L3 Morphology, Philadelphia Chromosome, MYC Gene Translocation, and Coexpression of TdT and Surface Light Chains: A Case Report

**DOI:** 10.1155/2013/679892

**Published:** 2013-03-06

**Authors:** Alicia C. Hirzel, Aaron Cotrell, Robert Gasparini, Vathany Sriganeshan

**Affiliations:** ^1^Mount Sinai Medical Center, A.M. Rywlin, Department of Pathology and Laboratory Medicine, 4300 Alton Road, Suite 2200, Miami Beach, FL 33140, USA; ^2^Neogenomics Laboratories, 12701 Commonwealth Drive, Fort Myers, FL 33913, USA

## Abstract

Acute lymphoblastic leukemia is predominantly found in children. It is a neoplasm of precursor cells or lymphoblasts committed to either a B- or T-cell lineage. The immature cells in B-acute lymphoblastic leukemia/lymphoma can be small or medium sized with scant or moderate cytoplasm and typically express B-cell markers such as CD19, cytoplasmic CD79a, and TdT without surface light chains. These markers, along with cytogenetic studies, are vital to the diagnosis, classification, and treatment of these neoplasms. We present an unusual case of a precursor B-cell ALL, in an 82-year-old woman, who presented with pancytopenia and widespread lymphadenopathy. The cells show L3 morphology (Burkitt-like lymphoma) with coexpression of TdT and surface light chains in addition to an MYC gene translocation and Philadelphia chromosome.

## 1. Introduction

Precursor lymphoid neoplasms are categorized as B lymphoblastic leukemia/lymphoma and T lymphoblastic leukemia/lymphoma. The B acute lymphoblastic leukemia/lymphoma (ALL) is further classified into B-ALL not otherwise specified or those with recurrent genetic abnormalities. As the name suggests, the cells that proliferate are immature lymphoid cells known as lymphoblasts, which have become halted in their development. Acute lymphoblastic leukemia/lymphoma is rare in the adult population, and the lymphoblasts that are characteristic of the disease show a spectrum of differentiation and have varied cytogenetic alterations. These lymphoblasts commonly have L1 and L2 morphology (FAB classification) with expression of TdT and other primitive lymphoid antigens. Rare cases of ALL with an atypical morphology, immunophenotype, and genetic makeup can pose a diagnostic challenge. We present an unusual case of a precursor B-cell ALL, in an 82-year-old woman, with L3 morphology (Burkitt-like lymphoma) that demonstrates coexpression of TdT and surface light chains. Additionally, the MYC gene translocation and Philadelphia chromosome were also present. Although cases of ALL with co-expression of TdT and surface light chains have been described, to our knowledge no published cases of ALL with this specific immunophenotype and genetic make-up have been reported in the literature. This case further illustrates the need for comprehensive immunophenotyping and genetic testing when establishing an accurate diagnosis of ALL, as this could impact management. Elderly patients with adverse cytogenetic abnormalities and high white blood cell count have a very poor prognosis.

## 2. Case Presentation

**Figure 1:**
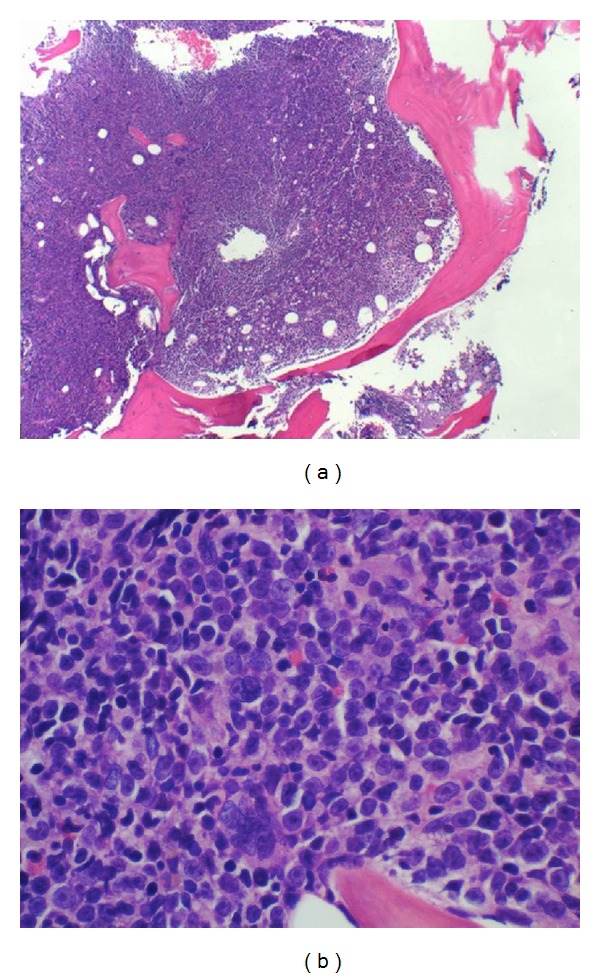
(a) Bone marrow biopsy, H&E stain, 4x image showing hypercellular marrow. (b) Bone marrow biopsy, 40x image showing large cells with prominent nucleoli.

**Figure 2:**
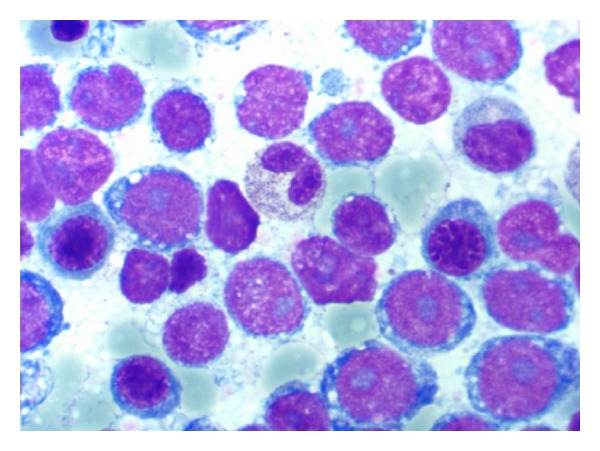
Aspirate smear, Wright-Giemsa stain, 100x image showing the neoplastic lymphoid cells with basophilic cytoplasm, cytoplasmic vacuoles, and large prominent nucleoli.

**Figure 3:**
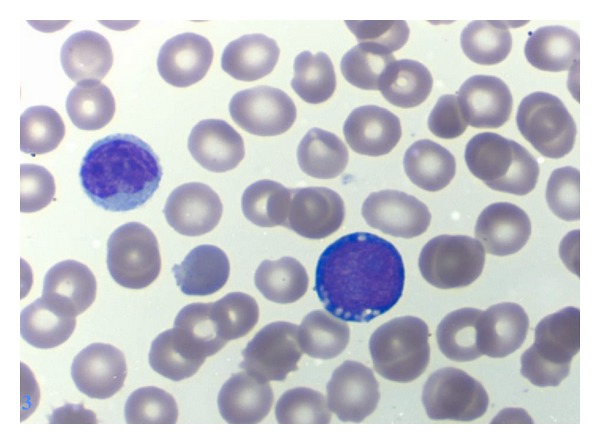
Peripheral smear, Wright-Giemsa stain, 100x image showing an abnormal circulating lymphoid cell with the same cytology as the lymphoid cells in the bone marrow.

**Figure 4:**
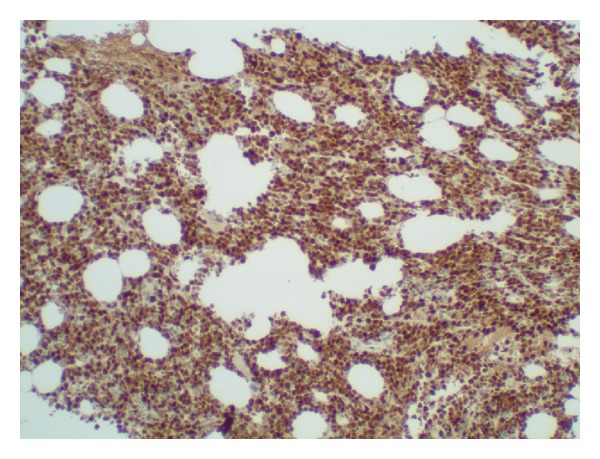
TdT staining abundant cells in bone marrow.

**Figure 5:**
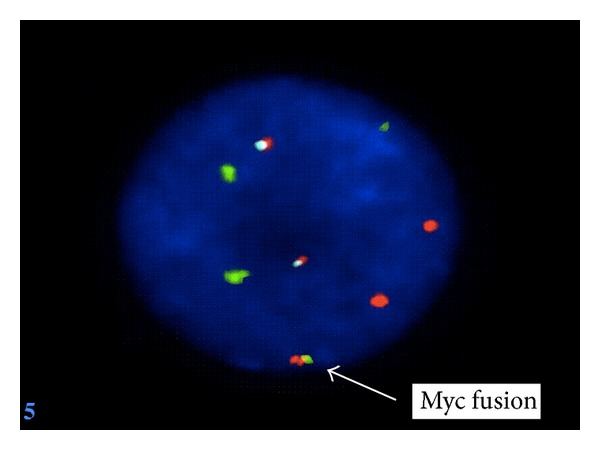
Interphase FISH analysis showing a normal chromosome 9 and 22, MYC gene translocation, and an extra red MYC signal translocated onto chromosome 7.

**Figure 6:**
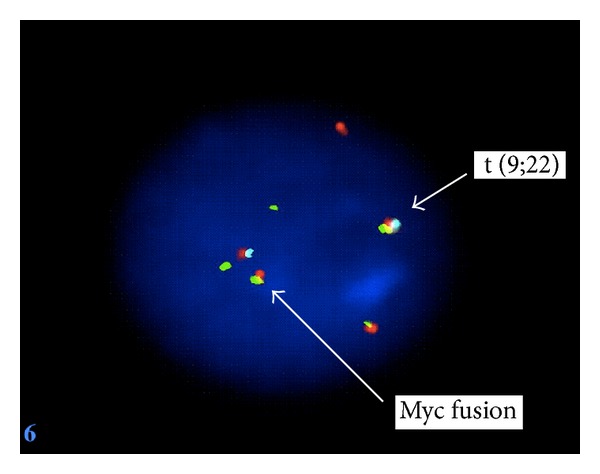
Interphase FISH analysis showing a t(9;22) and MYC gene translocation.

An 82-year-old women with a medical history significant for congestive heart failure and renal failure presented to the emergency room with shortness of breath. Laboratory studies revealed anemia (Hgb 9 g/dL), thrombocytopenia (52.0 × 10^3^/uL), white blood cell count of 6.0 × 10^3^/uL, and an elevated LDH (1800 U/L). Imaging studies demonstrated mediastinal, internal mammary, epicardial, and upper abdominal lymphadenopathy. A bone marrow biopsy was performed to evaluate the cause of the cytopenias and also to establish a probable correlation with the widespread lymphadenopathy. Bone marrow biopsy and aspirate revealed a hypercellular marrow almost completely replaced by a diffuse infiltrate of large lymphoid cells (Figures 1(a) and 1(b)) with basophilic cytoplasm, cytoplasmic vacuoles, and large prominent nucleoli (L3/Burkitt lymphoma-like morphology) (Figure 2). A peripheral smear showed few circulating abnormal lymphoid cells (5%) with the same cytologic features as the cells found in the bone marrow (Figure 3). Further characterization of the lymphoid cells using Immunohistochemical stains showed positivity for TdT (Figure 4), CD10, CD20, MUM-1, PAX5, and BCL 2. Stains for BCL-6 and CD34 were negative. Ki-67 showed a low proliferation index. The MUM1 and BCL2 were done using DAKO monoclonal mouse antibody provided in liquid form as cell culture supernatant dialysed against 0.05 mol/L Tris/HCL, pH 7.2, and containing 15 mmol/L NaN_3_. The TdT was done using CELL MARQUE Anti-TdT rabbit polyclonal antibody purified from rabbit anti-sera diluted in tris buffered saline, pH 7.3–7.7, with protein base, and preserved with sodium azide. Flow cytometry revealed a population of monoclonal B-cells (65%) expressing dim CD45, CD19, CD20, CD10, HLA-DR, kappa light chain, and bright CD38. Cytogenetic analysis revealed an abnormal female karyotype (46,XX,t(9;22)(q34;q11.2)[3]/46,XX[17]) with the Philadelphia chromosome. Interphase FISH analysis for t(9;22) and a MYC gene translocation revealed multiple genetic alterations including: a cell line with a normal chromosome 9 and 22 and concurrent MYC translocation; a cell line with a normal chromosome 9 and 22 and a MYC translocation with an extra copy of MYC translocated to chromosome 7; a cell line with both a t(9;22) and MYC translocation (Figures 4, 5, and 6).

## 3. Discussion

The WHO classification of tumors of hematopoietic and lymphoid tissues divides B-cell malignancies into precursor B-cell neoplasms and mature B-cell neoplasms. Precursor B cell leukemias are more frequent in the pediatric population, 75% of cases occurring in children under six years of age. While the rate of remission is high in both adults and children, the 5 year survival rate is much lower in adults. The French-American-British (FAB) classification was used prior to the new World Health Organization classification in 2008. The FAB classification was based on the morphology of the cells. Small round blasts with clumped chromatin were classified as L1. Larger blasts with nuclear pleomorphism and fine chromatin were classified as L2. L3 type morphology included all large blasts with nucleoli and vacuolated cytoplasm. Precursor B- and T-cell leukemias usually have L1 and/or L2 morphology, with expression of B- or T-cell antigens, respectively, and expression of TdT. They lack surface immunoglobulin expression. Nonetheless, some precursor B-cell leukemias can express cytoplasmic immunoglobulins. Precursor B ALL is known to have recurrent genetic abnormalities such as t(9:22)(q34;q11.2), t(v;11q23), t(12:21)(p13;q22), t(5;14)(q31;q32), t(1;19)(q23;p13.3), as well as hyperdiploid and hypodiploid. However, a MYC translocation, as well as others, is not unheard of in B-ALL.

Burkitt leukemia/lymphoma is now considered a mature B-cell neoplasm. It was formerly classified as precursor B ALL with L3 morphology (FAB-L3). The lymphoma cells are positive for B cell antigens, express surface immunoglobulins, and lack TdT expression with nearly 100% of cells being positive for ki-67. They all have translocations involving the c-myc gene located at 8q24.

 ALL is rare in adults and is commonly characterized by L1 or L2 morphology (FAB classification). TdT expression, lack of surface light chains, and variable genetic alterations are also common [1]. These features help distinguish between immature leukemia/lymphomas from more mature leukemia/lymphomas such as Burkitt lymphoma, diffuse large B-cell lymphoma, and the blastoid variant of mantle cell lymphoma. The distinction is important as the accurate diagnosis of an immature versus mature leukemia/lymphoma directs treatment.

The morphologic features in our case initially indicate a more mature differentiation (L3). Positivity for CD20, PAX5, and BCL 2, expression of surface kappa light chains and dim CD20 by flow cytometry again suggest a more mature lymphoma. However, the strong and diffuse expression of TdT, low proliferation index by ki-67, and bright CD38 by flow cytometry favor a diagnosis of an immature precursor B-cell leukemia/lymphoma. Coexpression of surface light chains and TdT has been previously reported in ALL [2, 3]. Presence of the Philadelphia chromosome, although described rarely in mature lymphomas [4], further supports the diagnosis of ALL and portends a worse prognosis. A MYC gene rearrangement is most frequently associated with Burkitt lymphoma yet has been reported in other mature and immature B-cell neoplasms [5, 6].

## 4. Conclusion

The isolated and concurrent translocations in the varied cell lines detected by FISH make this case more unique and difficult to explain. The questions posed include whether this case represents clonal evolution from a leukemic clone characterized by the immature immunophenotype and t(9;22) to a lymphoma-like clone with a mature immunophenotype and MYC translocation; or is this presentation the result of two different clones present from the beginning of the disease. Although there have been reports of concurrent t(9;22) and MYC translocations in pediatric cases of B-cell ALL, to our knowledge, no such cases in adults have been reported in the literature with this mature morphology, mixed immunophenotype, and complex genetic alterations.

## Abbreviations

TdT: Terminal deoxynucleotidyltransferase
WHO: World Health Organization
ALL: Acute lymphoblastic leukemia/lymphoma.
